# Acute Hepatic Failure as a Leading Manifestation in Exertional Heat Stroke

**DOI:** 10.1155/2012/295867

**Published:** 2012-06-28

**Authors:** Qi Jin, Erzhen Chen, Jie Jiang, Yiming Lu

**Affiliations:** Department of Emergency, Shanghai Rui Jin Hospital, Shanghai Jiao Tong University School of Medicine, Shanghai 20025, China

## Abstract

*Background*. Acute hepatic failure (AHF) is uncommon as a leading symptom in patients with exertional heat stroke (EHS). Which stage to perform the liver transplantation for severe hepatic failure in EHS is still obscure at clinical setting. The conservative management has been reported to be successful in treating heat-stroke-associated AHF even in the presence of accepted criteria for emergency liver transplantation. *Case Presentation*. Here, we reported a 35-year-old male who presented with very high transaminases, hyperbilirubinemia, significant prolongation of the prothrombin time, and coma. No other causes for AHF could be identified but physical exhaustion and hyperthermia. Although the current patient fulfilled London criteria for emergency liver transplantation, he spontaneously recovered under conservative treatment including intravenous fluids, cooling, diuretics as mannitol, and hepatocyte growth-promoting factors. *Conclusions*. Meticulous supportive management could be justified in some selected cases of AHF due to EHS.

## 1. Introduction

Exertional heat stroke (EHS) is a life-threatening condition caused by excess heat generated from muscular exercise that exceeds the body's ability to dissipate it at the same rate [1]. Potential complications of EHS include acute renal failure, acute hepatic failure (AHF), rhabdomyolysis, disseminated intravascular coagulation (DIC), and multiorgan dysfunction [2, 3].

While mild and moderate hepatic injury is a relatively common feature of EHS [4], few patients undergo fatal extensive hepatocellular damage [5, 6]. To date, no definite indications for liver transplantation to AHF in heat stroke have been established. Three patients with EHS experiencing liver transplantation died within one year [6–8], and one case was reported to survive for more than one year [9]. The conservative management has been described to be successful in treating heat-stroke-associated AHF even in the presence of accepted criteria for emergency liver transplantation [10, 11]. Recently, we experienced a patient who had AHF as a leading symptom during the course of EHS and who survived with the complete recovery of liver function under conservative treatment. Here we reported this unusual case with a review of literature.

## 2. Case Report

**Figure 1:**
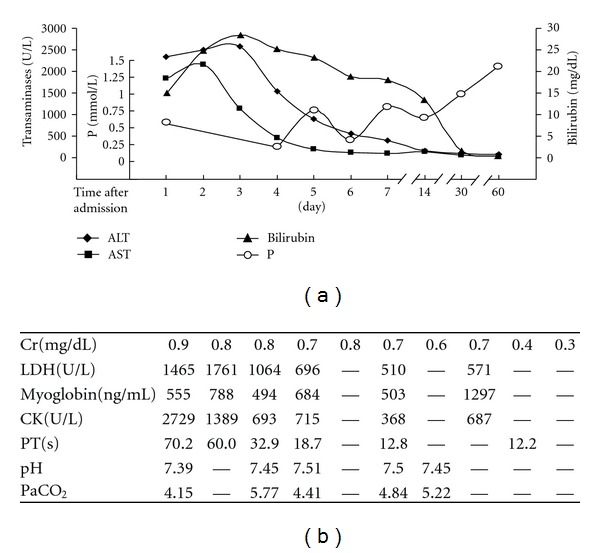
Basic laboratory parameters of the patient presenting with AHF during the course of EHS. In (a), ALT and AST began to be decreased from day 3 after admission in our ICU and recovered to be normal within 2 weeks. Total bilirubin declined more slowly than transaminases. In the early stage of EHS, hypophosphatemia was evident. (b) Showed the dynamic change of Cr, LDH, CK, PT and arterial blood gas analysis with pH and PaCO_2_ within 2 months in our hospital. ALT, alanine aminotransferase; AST, aspartate aminotransferase; P, phosphonium; Cr, creatinine; LDH, lactate dehydrogenase; CK, creatinine kinase; PT, prothrombin time.

 A previously healthy 35-year-old male was found unconscious after a 24 h consecutively physical work under heavy heat load and was delivered to the resuscitation room and our Intensive Care Unit of the Department of Emergency, Rui Jin hospital, Shanghai Jiao Tong University School of Medicine, 2 days later in July 2008. On admission, the patient had spontaneous respiration at the rate of 36 breaths per min, and his pulse was about 130 beats per min with regular rhythm. He was found to be comatose at grade 5 on the Glasgow Coma Scale (GCS; E1V1M3). The patient's surface temperature was more than 40°C. Complete blood count showed white blood cell 14,300/*μ*L, hemoglobin 13.9 g/dL, hematocrit 40%, platelet 19,000/*μ*L. The results of blood biochemistry exhibited very high transaminases, 2336 U/L alanine aminotransferase (ALT) (normal < 64 U/L), 1841 U/L aspartate aminotransferase (AST) (normal < 42 U/L), and significantly elevated total bilirubin 14.9 mg/dL (normal < 1.4 mg/dL) (Figure 1). Further laboratory parameters showed an evident prolongation of the prothrombin time (PT) (70.2 s, control 13 ± 3 s), D-dimer (1.22 mg/L, normal < 0.5 mg/L), an elevated lactate dehydrogenase (LDH) (1465 U/L, normal < 192 U/L), creatinine kinase (CK) (2729 U/L, normal < 269 U/L), and myoglobin (555 ng/mL, normal < 70 ng/mL) (Figure 1). His electrocardiogram showed ST elevation in lead I, II, III, aVL, aVF, and V1-5.

To exclude other causes for AHF, virus serological tests were performed.There were no positive findings for acute or chronic hepatitis A, B, C, E or human immunodeficiency virus (HIV). Also, acute infection with Epstein-Barr virus (EBV) and cytomegaly virus (CMV) was ruled out. The autoimmune antibodies (ANA, ANCA, ENA, RF) were negative. In addition, an abdominal ultrasound and CT scan did not exhibit the evidence of dilated bile ducts. After normalization of prothrombin time and platelet count, a liver biopsy was performed for exact staging of the severity of hepatic damage. Liver histology showed that the areas of liver cell necrosis contain a mild inflammatory infiltrate consisting of lymphocytes, plasma cells, and neutrophilic leukocyte. Liver cell vacuolization and fatty degeneration were present to differing extents. The immunohistochemical results indicated negative for HBsAg and HBcAg as well as negative HCV.

The patient was further monitored in our ICU and accepted the treatment of cooling that included cold saline infusion from gastric tube, ice cap, ice pack to axillae, neck and groin, and cold alcohol applied to the patient's skin. Meanwhile, we also administrated other supportive therapy with intravenous fluids, mannitol, and hepatocyte growth-promoting factors, and so on. The abnormal laboratory parameters returned slowly to normal within a few days while the renal function was always normal after admission (Figure 1). Because of the continual elevation of the ST segment in ECG and elevated myocardial enzymes, the echocardiography was performed and it indicated regional wall motion abnormality with a 43% ejection fraction.

The patient regained consciousness on day 7 from the onset of coma. He was delayed to be transferred to a regular ward because of toxic epidermis necrosis induced by vancomycin for treating respiratory tract infection caused by methicillin-resistant staphylococcus aureus. After the skin erosion disappeared and new epidermis covered the body, he was admitted to an internal medicine ward. Finally, at the time of discharge from our hospital, the patient's laboratory parameters had returned to normal values.

## 3. Discussion

In the present case study, we described a young patient with severe EHS that was mainly complicated with AHF as well as DIC, the failure of heart and central nervous system. The success in treating this EHS case suggested that the physicians and intensivists could consider appropriate supportive therapy to AHF as a predominant manifestation under the intensive investigation during the course of EHS.

The clinical manifestations of heat stroke are variable. Hyperthermia and central nervous system dysfunction must be present for a diagnosis of heat stroke. Hepatic injury in most cases of EHS is usually asymptomatic and can be reversed [4]. Approximately 5% of EHS experienced fulminant hepatic failure, which might be fatal [12]. Orthotopic liver transplantation (OLT) has been suggested as a potential therapy despite that even the extensive may recover spontaneously. However, to the best of our knowledge, the outcome of OLT in the four reported cases seemed to be disappointed. The first three patients underwent OLT on 8 days, 72 h, and 48 h respectively, after heat stroke and died of systemic infection, chronic transplantation rejection, and cardiopulmonary arrest respectively, within one year after liver transplantation [6–8]. Only one patient survived for more than one year after living donor liver transplantation [9]. Although the current patient fulfilled accepted London criteria [13] for emergency liver transplantation on day 2 after admission (PT longer than 50 s, bilirubin higher than 17.5 mg/dL, and non-A, non-B hepatitis (Figure 1)), we did not decide to perform liver transplantation immediately. The reasons that we decided upon watchful waiting for one more day are as follows: (1) while acute renal failure and acute respiratory distress are frequently seen in EHS, this patient did not have to receive the invasive mechanical ventilation and his plasma creatinine was always in the normal range; (2) the poor and limited outcome of liver transplantation in heat stroke in the previous case studies; (3) a previous study demonstrated, in the conservatively managed group of EHS-induced liver failure, 61.5% patients recovered spontaneously [11], (4) despite that the patient's PT was longer than 50 s, impaired coagulation tests were overestimated because of concomitant heat-induced endothelial injury and the consequent DIC.

EHS is a medical emergency that results in multiorgan dysfunction, which carries a high mortality. Very recently, a retrospective study [14] demonstrated that high levels of CK (>1000 U/L), metabolic acidosis, and elevated liver enzymes were predictive for multiorgan dysfunction among the various parameters during heat stroke. The overall case fatality rate was more than 70% and the mortality was even higher (85%) in patients with dysfunction of two or more organs. Accurate estimation of prognosis in AHF is a paramount goal. Evaluations of the prognostic criteria have had varied results; while some appear promising, more researches are needed to determine their reliability [15]. The predictor of AHF during EHS remains less clear. Recently, Gracin JM et al. reported that hypophosphatemia (<0.5 mmol/L) was the only independent predictive factor of AHF in confirmed EHS patients by multivariate analysis (RR 3.8, 95% CI 1.1–6.2). Consistent with Gracin's study, this patient appeared to be a marked hypophosphatemia that altered from 0.22 mmol/L to 0.81 mmol/L (normal 0.8–1.6 mmol/l) during the first two weeks after admission (Figure 1), although physiological phosphorus requirement was administrated each day. Hypophosphatemia has been observed consistently in patients with conditions characterized by fever or hyperthermia. The mechanism of hypophosphatemia in acute heat stroke is still elusive. The possible mechanisms are as follows. (1) Heat-stroke-related hypophosphatemia was associated with abnormal phosphaturia independent of the parathyroid hormone level. (2) Acute respiratory alkalosis induced by hyperthermia increased intracellular pH and caused phosphorus to shift from the extracellular to the intracellular compartment. (3) The elevation of body temperature increased intracellular utilization of phosphate in the glycolytic pathway, causing phosphorus to shift from the extracellular fluid into cells. In this patient, respiratory alkalosis presenting in the early stage of EHS could be one of the causes that resulted in hypophosphatemia. But it was unlikely to be the sole explanation for the observed hypophosphatemia because it sometimes occurred without respiratory alkalosis (Figure 1). However, to the best of our knowledge, there is no evidence that hypophosphatemia by itself could result in important liver dysfunction. Therefore, phosphatemia should be measured systematically on admission and 1-2 weeks later, and phosphorus should be supplied and evaluated as a predictor for AHF secondary to EHS.

In conclusion, appropriate supportive therapy in some cases could be justified in the early stage of AHF due to EHS and substantially reduce the mortality. It is now necessary to establish a scoring system for stratification of severity and prediction of mortality. Further clinical experience is needed to weigh the risk and benefit of conservative therapy or organ transplantation to treat AHF associated with heat stroke
